# Clone and functional analysis of Seryl-tRNA synthetase and Tyrosyl-tRNA synthetase from silkworm, *Bombyx mori*

**DOI:** 10.1038/srep41563

**Published:** 2017-01-30

**Authors:** Jingsheng Hu, Jianghai Tian, Fanchi Li, Bin Xue, Jiahuan Hu, Xiaoyu Cheng, Jinxin Li, Weide Shen, Bing Li

**Affiliations:** 1 School of Basic Medicine and Biological Sciences, Soochow University, Suzhou, Jiangsu 215123, P.R. China; 2 National Engineering Laboratory for Modern Silk, Soochow University, Suzhou, Jiangsu 215123, P.R. China

## Abstract

Aminoacyl-tRNA synthetases are the key enzymes for protein synthesis. Glycine, alanine, serine and tyrosine are the major amino acids composing fibroin of silkworm. Among them, the genes of alanyl-tRNA synthetase (AlaRS) and glycyl-tRNA synthetase (GlyRS) have been cloned. In this study, the seryl-tRNA synthetase (SerRS) and tyrosyl-tRNA synthetase (TyrRS) genes from silkworm were cloned. Their full length are 1709 bp and 1868 bp and contain open reading frame (ORF) of 1485 bp and 1575 bp, respectively. RT-PCR examination showed that the transcription levels of SerRS, TyrRS, AlaRS and GlyRS are significantly higher in silk gland than in other tissues. In addition, their transcription levels are much higher in middle and posterior silk gland than in anterior silk gland. Moreover, treatment of silkworms with phoxim, an inhibitor of silk protein synthesis, but not TiO_2_ NP, an enhancer of silk protein synthesis, significantly reduced the transcription levels of aaRS and content of free amino acids in posterior silk gland, therefore affecting silk protein synthesis, which may be the mechanism of phoxim-silking disorders. Furthermore, low concentration of TiO_2_ NPs showed no effect on the transcription of aaRS and content of free amino acids, suggesting that TiO_2_ NPs promotes silk protein synthesis possibly by increasing the activity of fibroin synthase in silkworm.

Silkworm (*Bombyx mori*) is one of the most important economic insects. The silk protein secreted by its silk gland is an important raw material for silk production. The contents of fibroin and sericin in silk protein are 70~75% and 25~30%, respectively^1^. Fibroin contains 20 kinds of amino acid residues, among which, glycine, alanine, serine and tyrosine residues account for 45.9%, 30.3%, 12.1% and 5.3%, respectively, while the other 16 amino acid residues account for only 6.5%^2^.

Aminoacyl-tRNA synthetases (aaRS), also known as aminoacyl-tRNA ligases, or amino acid activating enzymes, are enzymes that attach appropriate amino acid onto its tRNA in protein translation process^3^. aaRS specific for each of the 20 amino acids have been identified, and classified into two structurally distinct and apparently unrelated classes, each encompassing 10 specificities^4^,^5^. Wherein, SerRS belongs to the Class II aaRS and TyrRS belongs to the Class I aaRS^6^,^7^. Thus, aaRS for glycine, alanine, serine and tyrosine play important roles in fibroin synthesis. Nada S *et al*.^8^ and Chang P K *et al*.^9^ have cloned and analyzed GlyRS and AlaRS, respectively, but SerRS and TyrRS have not been studied and reported yet.

In recent years, large-scale abuse of phoxim pesticide has significantly affected mulberry, leading to pesticide poisoning of silkworm, and significantly restricting the development of sericulture^10^,^11^. For example, synthetic phoxim has been shown to impede fibroin synthesis, resulting in a decline of silk production of silkworm^12^,^13^. TiO_2_ NPs, as a new additive, can not only improve silk production of silkworm^14^,^15^, but also alleviate phoxim-caused metabolic disorders of silk gland^13^,^16^. In this study, SerRS and TyrRS were cloned and the effects of TiO_2_ NPs and phoxim treatments on the expression levels of SerRS, TyrRS, AlaRS and GlyRS were analyzed.

## Results

### Clone of full-length cDNA of SerRS and TyrRS

**Figure 1:**
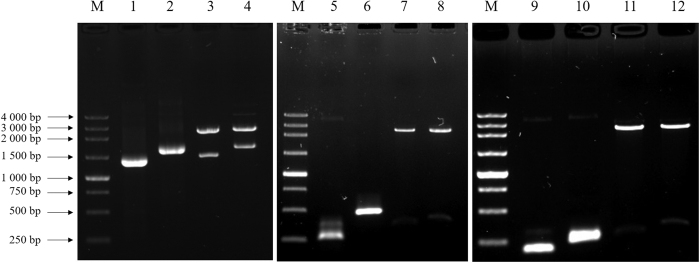
Identification of TyrRS and SerRS PCR products and and recombinant plasmids using restriction enzyme digestion. M: 250 bp DNA marker; 1–2: Positive controls of SerRS and TyrRS PCR products; 3–4: restriction enzyme digestion of SerRS and TyrRS PCR products; 5–6: 5′ UTR positive controls of SerRS and TyrRS; 7–8: restriction enzyme digestion of SerRS and TyrRS 5′ UTR; 9–10: 3′ UTR positive controls of SerRS and TyrRS; 11–12: restriction enzyme digestion of SerRS and TyrRS 3′ UTR.

**Figure 2:**
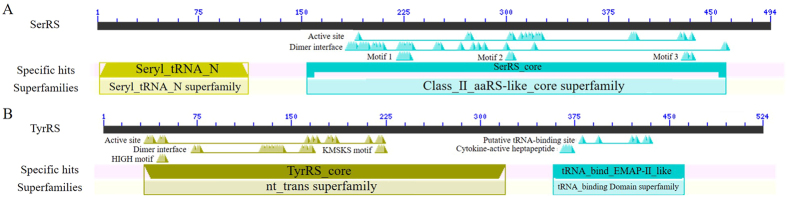
Functional analysis of Structure domains of SerRS (**A**) and TyrRS (**B**).

The full-length SerRS and TyrRS genes were cloned using cDNA from silk gland as template by RT-PCR and RACE technology. First, a 1655 bp SerRS fragment and a 1374 bp TyrRS fragment were amplified by RT-PCR. Then the 269 bp and 371 bp 5′ UTR as well as 229 bp and 242 bp 3′ UTR of SerRS and TyrRS were amplified using 5′ RACE and 3′ RACE techniques, respectively (Fig. 1). After splicing, the full-length sequences of 1709 bp SerRS (Fig. 2A) and 1868 bp TyrRS of silkworms (Fig. 2B) were obtained (NCBI No. KU955848, KU955849).

### Evolutionary analysis of SerRS and TyrRS

**Figure 3:**
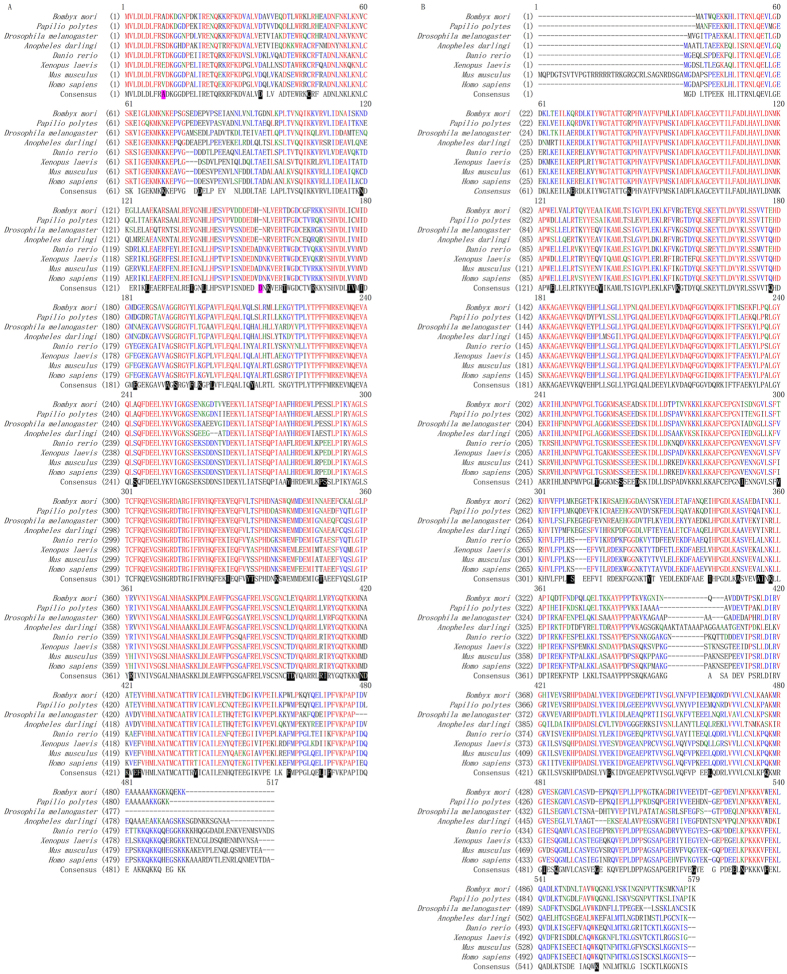
Sequence comparison of SerRS (**A**) and TyrRS (**B**) from multi-species. The blackened residues indicate characteristic amino acids were potential species recognition sites.

Amino acid sequence of SerRS showed species specificity. Its 10^th^ amino acid residue is alanine in Lepidoptera, serine in Diptera, and valine in mammals (Fig. 3A); its 34^th^ amino acid residue is aspartate in all species but Diptera, which is glutamine residue; its 400^th^ amino acid residue is leucine in insects, but threonine in vertebrates; its 213^rd^ and 282^nd^ amino acid residues vary randomly among three amino acid residues; and the sequences at its 258–267 positions are highly conserved in all vertebrates. In TyrRS, the sequences of amino acid residues at 75–123 and 167–230 positions are highly conserved and show no significant differences among species besides random substitution of among several amino acid residues (Fig. 3B). By contrast, the sequences of amino acid residues between 388 and 413 positions are not conserved in insects, but relatively conserved in vertebrates. Moreover, the amino acid sequences in the c-terminal half of TyrRS are not highly homologous.

**Figure 4:**
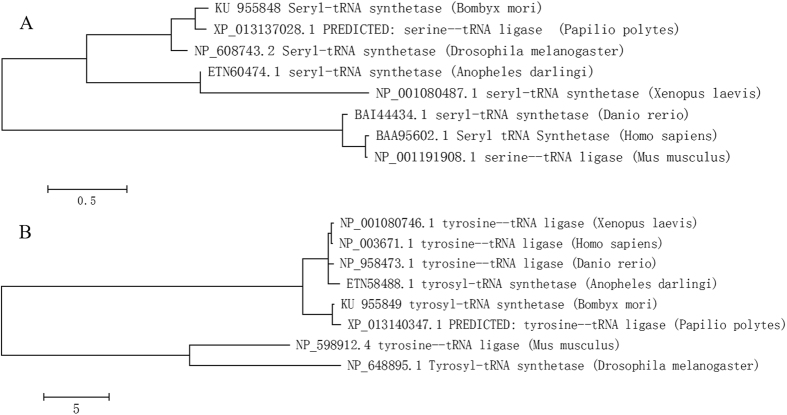
Phylogenetic tree analysis of SerRS and TyrRS. (**A**) Phylogenetic tree of SerRS, (**B**) Phylogenetic tree of TyrRS.

SerRS and TyrRS sequences of silkworm were compared with those of 7 other insect species on the NCBI database using MEGA6.0 software to analyze their evolutionary relationship. Phylogenetic analysis showed that the cloned genes had high homology to lepidopteran insects (Fig. 4), wherein silkworm SerRS and TyrRS had the closest relationship with *Papilio polytes*, sharing 87% and 82% homology, respectively.

### Expression of SerRS, TyrRS, AlaRS and GlyRS in silkworm tissues

**Figure 5:**
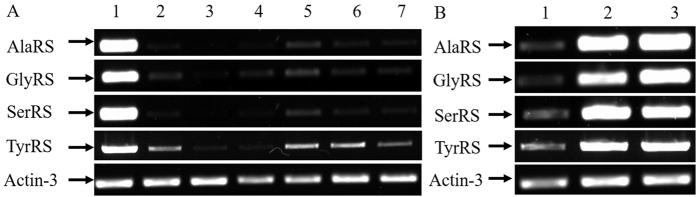
Specific expression of SerRS, TyrRS, AlaRS and GlyRS in silkworm tissues. (**A**) Expression of SerRS, TyrRS, AlaRS and GlyRS in different tissues of silkworms. 1: silk gland; 2: midgut; 3: fat body; 4: Markov pipe; 5: Head; 6: gonads; 7: hemolymph. (**B**) Expression of SerRS, TyrRS, AlaRS and GlyRS in different parts of silk gland. 1: anterior silk gland, 2: middle silk glands; 3: posterior silk gland.

RT-PCR analysis was used to detect the transcription levels of SerRS, TyrRS, AlaRS and GlyRS in 7 tissues of silkworm. The results showed that mRNA level of these aaRS was the highest in the silk gland (Fig. 5A). To further study their expression profiles in the silk gland, their expression in anterior, middle and posterior silk gland was examined. RT-PCR showed that SerRS, TyrRS, AlaRS and GlyRS were highly expressed in the middle and posterior silk gland (Fig. 5B).

### Effects of TiO_2_ NPs and phoxim treatments on expression of aaRS and content of amino acid in silk gland

**Figure 6:**
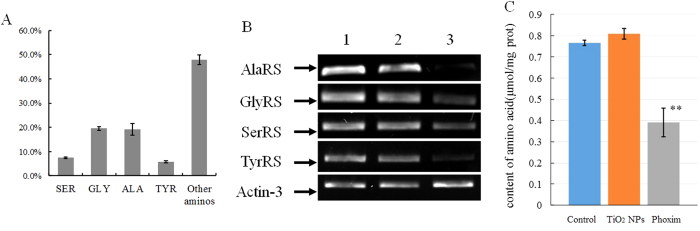
Effect of TiO_2_ NPs treatment and phoxim treatment in silk gland. (**A**) The component of silk protein in silk gland. (**B**) Transcription levels of SerRS, TyrRS, AlaRS and GlyRS in silk gland. 1: control; 2: TiO_2_ NPs; 3: phoxim. (**C**) Fold change of free amino in silk gland. Treatments with different letters indicate significantly different values (*P < 0.05, **P < 0.01), Values represent means ± SD, n = 3.

The component of silk protein in silk gland showed that content of serine, tyrosine, alanine and glycine was 7.39%, 5.80%, 19.24% and 19.54%, respectively, and other amino acids was 47.93% in total. RT-PCR analysis showed that the transcription levels of SerRS, TyrRS, AlaRS and GlyRS in the silk gland were not significantly affected by TiO_2_ NPs treatment, but significantly down-regulated by phoxim treatment (Fig. 6B). Free amino acid content in silk gland was not significantly affected by phoxim treatment and TiO_2_ NPs treatment (Fig. 6C), showing 1.013-fold in the TiO_2_ NPs group and 0.740-fold in the phoxim group.

## Discussion

All aaRS can be divided into Class I and Class II. The former includes MetRS, ValRS, IleRS, LeuRS, CysRS, GluRS, GlnRS, LysRS, ArgRS, TrpRS and TyrRS, and the latter includes AlaRS, HisRS, ProRS, ThrRS, SerRS, GlyRS, PheRS, AspRS, AsnRS and LysRS^17^. Previous research showed that TyrRS contains two functional domains, namely the N-terminal catalytic core and C-terminal EMAP II^18^. This study showed that silkworm TyrRS is composed of the catalytic core domain and tRNA-binding EMAP II domain (Fig. 2B). Among them, the catalytic core domain contains HIGH and KMSKS motifs, the two motifs of Class I aaRS^19^. In the Lepidoptera, HIGH motif and KMSKS motif composes the active site of the catalytic core domain. SerRS contains a special spiral arm structure at the N-terminus and plays an important role in catalyzing aminoacylation^20^. In addition, its motif 1 locates at the dimer interface, while the motif 2 and motif 3 locate in the active site of the catalytic core domain^21^. Sequencing analysis showed that the catalytic core domain of silkworm SerRS belongs to Class II aaRS and contains three characteristic motifs (Fig. 2A). The 10^th^ amino acid residue in the N-terminal conserved region of SerRS is alanine in Lepidoptera, serine in Diptera, valine in mammals and threonine in Cypriniform and glutamine in Anura, suggesting it could be a potential species recognition site (Fig. 3A). In addition, the 154^th^ amino acid residue is a stable and preserved insertion of aspartate in species with revolutionary position higher insects, inferring that the site is likely preserved in the evolution process by natural selection. The sequences of amino acid residues at 388–413 of TyrRS are not conserved in insects, but relatively conserved in vertebrates, suggesting that they are gradually stabilized and retained in the process of evolution (Fig. 3B). Moreover, the amino acid sequences in the c-terminal half of TyrRS are not highly homologous, promoting that this is due to the need of recognizing different tRNA in different species.

About 56% of the crystalline portion of silk fibroin was Ala-Gly-Ser-Gly-Ala-Gly repeat, while the remaining 44% of the non-crystalline portion is Tyr-rich region^22^. Thus, Ala, Gly, Ser and Tyr residues are important component of silk fibroin, and their corresponding aaRS play important roles in fibroin synthesis. The function of aminoacyl-tRNA synthesis is to precisely match amino acids with tRNAs containing the corresponding anticodon^23^. The role of aminoacyl-tRNA synthetases in translation is to define the genetic code by accurately pairing cognate tRNAs with their corresponding amino acids^24^. The present study found by RT-PCR that silk gland had the highest transcription levels of AlaRS, GlyRS, TyrRS and SerRS (Fig. 5A). Analysis of the components of silk protein in silk gland showed that the content of serine, tyrosine, alanine and glycine was 7.39%, 5.80%, 19.24% and 19.54%, respectively (Fig. 6A). As the vital organ for fibroin synthesis, silk gland is divided into the anterior, middle and posterior parts. Among them, the latter two parts are the major part of silk protein synthesis^25^. Consistently with previous studies, RT-PCR analysis of the present study showed that these two parts also had the highest transcription levels of AlaRS, GlyRS, TyrRS and SerRS (Fig. 5B), further confirming that higher expression of the four aaRSs as the main raw material providers for fibroin synthesis is required for large amounts of silk fibroin synthesis.

Silk gland is an important organ in silkworm for silk fibroin synthesis, but phoxim poisoning can cause metabolic abnormalities of silk gland, affecting fibroin synthesis and significantly reduced silk production^26^,^27^. The research showed that phoxim treatment significantly reduced the transcription levels of AlaRS, GlyRS, TyrRS and SerRS (Fig. 6B), suggesting that phoxim can decrease AlaRS, GlyRS, TyrRS and SerRS synthesis in silk gland, leading to fibroin synthesis disorders and reduced fibroin synthesis. Studies have shown that supplementing TiO_2_ NPs in silkworm feed can promote synthesis of Fib-H, Fib-L, P25, Ser-2 and Ser-3 in silkworm and eventually increase silk production in silkworm^15^,^16^,^28^. Meanwhile, supplementing TiO_2_ NPs can increase the content of free amino acids in haemolymph^29^. In this study, TiO_2_ NPs did not significantly affect the contents of free amino acids (Fig. 6C), and transcription levels of AlaRS, GlyRS, TyrRS and SerRS, which providing amino acid for fibrion synthesis. It suggests that TiO_2_ NPs promoted silk protein synthesis not by enhancing aaRS transcription, but by increasing the activity of fibroin synthase. Moreover, phoxim lead to reduction of not only aaRS transcriptional level, but also free amino acids level.

In summary, in the present study, we cloned and sequenced the full-length of silkworm TyrRS and SerRS, verified the results by domain and phylogenetic analyses (Fig. 4). The 10^th^ amino acid residue in the N-terminal conserved region of SerRS may be a potential species recognition site. In addition, we found that middle and posterior silk gland had the highest expression levels of AlaRS, GlyRS, TyrRS and SerRS, meeting the needs of fibroin protein synthesis. Moreover, we found that phoxim reduces silk fibroin synthesis by inhibiting transcription of aaRS and content of free animo, while TiO_2_ NPs promotes silk protein synthesis possibly by increasing the activity of fibroin synthase, but not by affecting the transcription levels of AlaRS, GlyRS, TyrRS and SerRS.

## Materials and Methods

### Materials

Silkworm variety Jingsong × Haoyue was preserved in our laboratory. TiO_2_ NPs with analytical purity of 99.99% were from Sangon Biotech (Shanghai) Co., Ltd (China). Phoxim [O, O-diethyl O- (alpha-cyanobenzylideneamino) phosphorothioate with purity of 98.1% was from Sigma-Aldrich.

### Insects and feeding

Silkworms were reared in the laboratory under 12 h light/12 h dark conditions to the fifth instar larvae. TiO_2_ NPs were formulated to 5 g/L stock solution and diluted to 5 mg/L working solution^16^,^30^. Phoxim was prepared as 100 g/L aceton solution, and diluted with double-distilled water to 4 mg/L working solution^31^. All treated mulberry leaves were dried naturally at room temperature and used to feed silkworm.

The fifth instar silkworms were divided into control, TiO_2_ NPs and phoxim groups with 100 in each group. Silkworms in the former two groups were fed with water, and 5 mg/L TiO_2_ NPs-treated mulberry leaves three times per day, respectively. Silkworms in the latter group were continuously fed with phoxim-treated mulberry leaves at the third day for 24 h. All silkworms were sacrificed, and their hemolymph, silk gland, head, fat body, midgut, Malpighian tubules and gonadal tissues were collected and stored at −80 °C.

### Gene cloning

The primers for cloning BmSer and BmTyr genes were designed based on the sequences of XM_004922403.1 and XM_004929703.1, respectively, and are listed in Table 1. The obtained sequences were blasted against NCBI database and confirmed to be correct. Then 3′ RACE and 5′ RACE primers were designed and used in RACE amplification. The first strand cDNA obtained using 3′ RACE was synthesized using 3′ anchor oligo ACGCTACACGACTCACTAATGGGC(T)_12_N^32^, and then amplified using nested PCR with gene specific primer and 3′ RACE M primer. 5′ RACE was performed using 5′-Full RACE Kit with TAP kit from Takara (Dalian) following the instructions provided by the manufacturer. All PCR products were cloned into pMD-18T vector (2 692 bp) and the obtained recombinant plasmids were analyzed by restriction enzyme digestion and sequencing performed by Suzhou Synbio-Tech Company.

### Sequence analysis

Sequencing results were confirmed against NCBI database and their protein domains were analyzed. All sequences were edited and analyzed using DNAMAN 8.0 software and the phylogenetic analysis was performed using MEGA 6.0 software. The sequences were aligned against those of other species (*Drosophila melanogaster, Danio rerio, Homo sapiens, Anopheles darling, Mus musculus, Xenopus laevis, Papilio polytes*).

### Total RNA extraction and RT-PCR

RNA was extracted from hemolymph, silk gland, head, fat bodies, midgut, Malpighian tubules and gonad of silkworms using the RNAiso kit (Takara, Dalian) and reverse transcribed into cDNA using the M-MLV RTase cDNA synthesis kit (TakaRa, Dalian).

PCR Primers for *Actin-3* were designed as previously reported^15^. RT-PCR primers specific for SerRS, TyrRS, AlaRS and GlyRS were designed based on the data from NM_001043987.1 and NM_001046828.1 of NCBI database (Table 1). The expression levels of SerRS, TyrRS, AlaRS and GlyRS in various tissues of control, phoxim treated and TiO_2_ NPs treated silkworms were investigated by PCR using cDNA as a template and *Actin-3* as the internal control.

### Measurement of total amino acid content

The levels of free amino acids in the silk gland were detected using the total amino acid content kit from KeMing (Suzhou) biotechnology Co Ltd. The component of silk protein in silk gland was detected by ecological textile dyestuffs and chemicals Testing Center (Zhejiang).

## Additional Information

**How to cite this article**: Hu, J. *et al*. Clone and functional analysis of Seryl-tRNA synthetase and Tyrosyl-tRNA synthetase from silkworm, *Bombyx mori. Sci. Rep.* **7**, 41563; doi: 10.1038/srep41563 (2017).

**Publisher's note:** Springer Nature remains neutral with regard to jurisdictional claims in published maps and institutional affiliations.

## Figures and Tables

**Table 1:** Primer sequences used in PCR.

Primer name	Primer sequences (5′-3′)	Primer size (bp)
BmSerRS-S	GACTTAGACTTATTTCGTGCCG	22
BmSerRS-A	AAGCCAAGGTTTGAGGATTT	20
BmTyrRS-S	CATACATCATTCAGCTTGATGC	22
BmTyrRS-A	CTCAGAAATGCACATAATGGA	21
5′race Ser outer	AGCCACTGGGTTCCTTGTTCTTC	23
5′race Ser inner	CTCCACAAGGTGTCTTGCTCGA	22
3′race Ser outer	CACTGAATACGTGCACATGCTGA	23
3′race Ser inner	CCATACTCGAAGTACACCAGACA	23
5′race Tyr outer	TGAGTGCGAAGTGCTACAAGCTC	23
5′race Tyr inner	TAGTAACCTCACAGCCAGCCTTC	23
3′race Tyr outer	ATGACACTGGTGAACCTGATGAG	23
3′race Tyr inner	CAGCGGTCTGGCAAGGTAATAAG	23
3′race M	ACGCTACACGACTCACTAATGGGCTT	26
Actin3-S	AACACCCCGTCCTGCTCACTG	21
Actin3-A	GGGCGAGACGTGTGATTTCCT	21
RTAla-S	CAGATGGTGGTTGCCCTGAT	20
RTAla-A	ATCAAGTCTTCCTGGCCTGC	20
RTGly-S	CTAAGGCCCGAAACAGCTCA	20
RTGly-A	TGCCCATGTGCTGTCTGAAT	20
RTSer-S	CTGCATGAGTCTGTACCAGT	20
RTSer-A	TCTGAAGCATGTGGAGAGTC	20
RTTyr-S	CCGCCAACAAAGGTCAAAGG	20
RTTyr-A	ATTACCTTGCCAGACCGCTG	20
